# Red Cell Phenotyping of Rh and Kell in Voluntary Blood Donors at a Tertiary Care Center in Jamnagar

**DOI:** 10.7759/cureus.79212

**Published:** 2025-02-18

**Authors:** Digeet P Davad, Rohitkumar P Panucha, Ketul J Patel, Jay Nagda

**Affiliations:** 1 Department of Immunohematology and Blood Transfusion, Shri M P Shah Government Medical College, Jamnagar, IND; 2 Department of Pathology, Shri M P Shah Government Medical College, Jamnagar, IND; 3 Department of Community Medicine, Shri M P Shah Government Medical College, Jamnagar, IND

**Keywords:** alloimmunization, extended phenotyping, haemolytic transfusion reactions (htrs), rh and kell antigens, voluntary blood donors

## Abstract

Background

The Rh and Kell blood group systems are among the most clinically significant in transfusion medicine due to their immunogenic potential. Alloimmunization of these antigens can lead to hemolytic transfusion reactions and hemolytic disease in the fetus and newborn. Despite their clinical importance, antigen phenotyping beyond ABO and RhD is limited in many blood centers worldwide, including in India. This study aims to determine the prevalence of Rh subgroups and Kell antigens among voluntary blood donors in Jamnagar to enhance transfusion safety.

Methods

This prospective observational study was conducted at Shri M P Shah Government Medical College and S P Mehta Blood Center in Jamnagar, India, from September to October 2022. A total of 1,000 voluntary in-house blood donors were included based on standard donation criteria. Blood samples were collected in EDTA vacutainers for phenotyping. Extended Rh (C, c, E, e) and Kell (K) antigen typing was performed using an automated platform (Immucor NEO, Immucor, Inc., Norcross, GA, USA), while the k antigen was assessed using the tube test method.

Results

Among the donors, RhD positivity was observed in 92.2% of samples. The most prevalent Rh antigens were e (99.67%) and C (93.06%), while E (18.66%) was the least common. The Kell (K) antigen was detected in 2.1% of donors. These findings are consistent with data from other Indian studies, with minor regional variations. The study also emphasizes the importance of establishing a donor database for antigen-negative units to effectively manage alloimmunized patients.

Conclusions

This study underscores the importance of extended phenotyping for Rh and Kell antigens in voluntary blood donors. Establishing a comprehensive donor database can greatly enhance transfusion safety, particularly for patients requiring repeated transfusions or those with alloantibodies. Further research and resource allocation are crucial for implementing widespread phenotyping, especially in resource-limited settings.

## Introduction

In the 17th century, physicians such as Richard Lower and Jean-Baptiste Denys performed blood transfusions between animals and humans, which resulted in severe reactions and fatalities due to blood incompatibility [1]. The 1901 discovery of the ABO blood group system by Landsteiner [2] marked a milestone in the modern understanding of blood group systems.

The primary objective of blood transfusion is to provide red blood cells from compatible donors, ensuring the highest possible survival rate in the recipient. Donor units are selected based on compatibility with the recipient’s red blood cells, which is determined by the presence or absence of specific antigens in these cells [3,4].

Red cell antigens, hereditary substances found on the surface of red blood cells, collectively form the “blood group system” [5]. These antigens exhibit variation across races and ethnicities, leading to the formation of alloantibodies when an individual's red cell antigens are exposed to foreign antigens through blood transfusions or pregnancy. This immune response is known as alloimmunization [6,7].

Alloimmunization is most commonly associated with alloantibodies such as Rh, Kell, Kidd, and Duffy. These antibodies react at 37°C, making them clinically significant. They can lead to hemolytic transfusion reactions (HTRs), both acute and delayed, as well as hemolytic disease of the fetus and newborn [8-10].

The rising frequency of alloantibodies beyond the ABO system is linked to the increasing demand for blood transfusions, driven by chronic illnesses and complex medical procedures [7,11]. Consequently, identifying suitable blood units for transfusion becomes more challenging [12]. To avoid potentially fatal incompatible transfusions, pre-transfusion testing and antigen phenotyping are crucial [13].

The Rh blood group system is one of the most complex and immunogenic, while the Kell system, discovered in 1946, is the third most potent in causing HTRs. Four nomenclature systems for Rh have been described: (1) Fisher-Race, (2) Wiener, (3) Rosenfield, and (4) International Society of Blood Transfusion (ISBT). The ISBT has officially recognized 349 antigens, which are organized into 36 blood group systems, nine of which are considered major systems [14,15].

Antigen phenotyping is especially important for patients requiring frequent transfusions, such as those with thalassemia, pregnant women at risk of alloimmunization, and individuals who have already become alloimmunized. However, in India, antigen phenotyping in most blood centers is typically limited to ABO and RhD typing. Expanding antigen typing to include other clinically significant systems is essential for selecting compatible blood units for alloimmunized patients [16-18].

Currently, the availability of compatible blood for these patients in India relies on cross-matching open inventory units, unlike in developed countries where donor phenotyping is either routine or mandatory for patients requiring multiple transfusions. The high financial cost of comprehensive antigen phenotyping limits its implementation in many Indian blood centers [19,20].

Understanding the prevalence of Rh subgroups and Kell antigens within a population can help create a donor database, reduce the risks of alloimmunization, and improve transfusion outcomes [21].

This study was conducted at our tertiary care center to assess the prevalence of Rh subgroups and Kell antigens among voluntary blood donors, comparing the results with data from other regions in India and different ethnic groups. The study aims to evaluate the prevalence of Rh subgroups and Kell antigens in the Jamnagar population and establish a database for identifying antigen-negative blood units for alloimmunized patients.

## Materials and methods

This prospective observational study was conducted at the Department of Immunohematology and Blood Transfusion, Shri M P Shah Government Medical College and S P Mehta Blood Center in Jamnagar, India, from September 7, 2022, to October 18, 2022.

The study included 1,000 voluntary in-house blood donors who met the inclusion criteria. Eligible donors were aged between 18 and 65 years, weighed over 50 kg, had a minimum hemoglobin level of 12.5 g/dL, and maintained a three-month interval between consecutive blood donations. Only individuals who fulfilled all pre-donation requirements were considered for participation.

Exclusion criteria included apheresis, professional donors, autologous donors, and those from blood donation camps. Additional exclusions were individuals with hemoglobin levels below 12.5 g/dL, liver disease, epilepsy, insulin-dependent diabetes, asthma, tuberculosis, or other chronic illnesses. Donors on medications for cardiovascular or hypertensive conditions, individuals testing positive for HBsAg, HCV, syphilis, or malaria, those with high-risk behaviors, self-injected drug users, pregnant women, transplant recipients, and individuals with significant weight loss (over 10% in the past month) were also excluded.

After confirming eligibility and obtaining informed consent, blood samples were collected in EDTA and plain vacuettes. EDTA samples were used for ABO grouping and antigen phenotyping. Extended Rh (C, c, E, e) and Kell (K) antigen phenotyping was performed using the automated Immucor Gamma Neo system (Immucor, Inc., Norcross, GA, USA), while the k antigen was analyzed using the tube test method according to the manufacturer’s instructions.

For laboratory procedures, a 5% red cell suspension was prepared by adding 50 µL of packed red cells to 1 mL of 0.9% normal saline in a labeled test tube, followed by thorough mixing. Serum blood grouping required pooled cell suspensions, which were prepared by washing three to four non-reactive samples of known blood groups (A, B, O) with normal saline three times, centrifuging to remove the supernatant, and resuspending the cell button in 5 mL of LISS to create a 5% pooled cell suspension.

ABO grouping was conducted using both forward and reverse methods. In forward grouping, two drops of anti-A, anti-B, and anti-AB monoclonal antisera were added to test tubes containing one drop of the 5% red cell suspension. The tubes were incubated at room temperature for 15-30 minutes, centrifuged at 1,000 rpm for one minute, and then examined visually and microscopically for agglutination. In reverse grouping, two drops of the donor’s serum were added to test tubes containing one drop of pooled cell suspensions (A, B, O), followed by the same incubation, centrifugation, and agglutination assessment (Table 1, Table 2).

**Table 1:** Interpretation of blood group

Reaction with red cell			Reaction of test serum with pooled cell			Interpretation of blood group
Anti-A	Anti-B	Anti-D	A	B	O	
+	+	+	0	0	0	AB
+	0	+	0	+/H	0	A
0	+	+	+/H	0	0	B
0	0	0	+/H	+/H	0	O
0	0	0	+/H	+/H	+	Oh (Bombay blood group)

**Table 2:** Reaction under microscopic examination

Reaction	Observation in a test tube
4+	Solid clumps
3+	Sever large clumps
2+	Small- to medium-sized clumps with a clear background
1+	Small clumps with a cloudy background
+Weak	Tiny aggregates with a cloudy background
Hemolyzed	No red cells in the test tube
Negative	No agglutination

Rh typing was performed using monoclonal anti-D (IgM) antisera, with both positive and negative controls included to ensure accuracy. Weak D testing was conducted for Rh-negative samples using the indirect antiglobulin test. Kell (k-micro) antigen testing was carried out with anti-k antisera using the tube test method, also incorporating positive and negative controls for validation.

Automated Rh phenotype analysis (C, c, E, e) was performed using monoclonal IgM Anti-D (IgG+IgM) from Immucor Gamma Neo (Immucor, Inc.) and anti-human globulin reagents. The results closely matched manual serological testing, demonstrating excellent accuracy and reproducibility. Automation reduces inter-operator variability, enhances standardization, and improves efficiency in high-throughput clinical laboratories. Precise Rh typing is essential in transfusion medicine to prevent alloimmunization and HTRs. The automated system ensures faster turnaround times while maintaining stringent quality control. Integrating into modern immunohematology workflows minimizes human error and streamlines blood bank operations. These findings underscore the value of automated Rh typing as a reliable, efficient, and standardized method for blood group phenotyping, improving patient safety and transfusion compatibility.

To ensure accuracy and prevent false positives or negatives, controls were included for all tests. Known positive and negative samples for specific antigens were used as controls, and all reagents and equipment were quality-tested following standard operating procedures.

Statistical analysis was performed by calculating the frequencies of antigens and phenotypes as absolute values and percentages. The results were presented using Microsoft Excel 2007 (Microsoft Corporation, Redmond, WA, USA) for graphical representation, ensuring accurate and reliable data collection for assessing the prevalence of Rh subgroups and Kell antigens in the study population.

## Results

This prospective study included 1,000 voluntary blood donors from Jamnagar to assess the prevalence of Rh subgroups and Kell antigens. The findings were analyzed and presented using tables and microscopic imaging.

Among the 1,000 donors, 997 were male (99.7%) and three were female (0.3%). The most common blood group was B, with 342 donors (34.2%), followed by O with 330 donors (33%), A with 218 donors (21.8%), and AB with 110 donors (11%). Rh(D) antigen testing revealed that 922 donors (92.2%) were Rh(D) positive, while 78 donors (7.8%) were Rh(D) negative (Table 3).

**Table 3:** Gender-wise, blood group-wise, and Rh status-wise distribution of donors (n = 1,000)

Category	Number of donors	Frequency (%)
Gender		
Male	997	99.7%
Female	3	0.3%
Blood group		
A	218	21.8%
B	342	34.2%
AB	110	11.0%
O	330	33.0%
Rh status		
D positive (D+)	922	92.2%
D negative (D-)	78	7.8%

Among Rh-positive donors, the B blood group was the most common (34.70%), while the O blood group was predominant (38.46%) among Rh-negative donors (Table 4).

**Table 4:** Distribution of ABO and Rhesus blood groups (n = 1,000)

Blood group	Rh positive		Rh negative		Total
	Number	Frequency (%)	Number	Frequency (%)	
A	201	21.80%	17	21.79%	218
B	320	34.70%	22	28.20%	342
AB	101	10.95%	9	11.53%	110
O	300	32.54%	30	38.46%	330
Total	922	100%	78	100%	1,000

Among Rh(D)-positive donors, the most frequently detected antigen was e (99.67%), followed by C (93.06%), c (56.39%), and E (18.66%). In contrast, Rh(D)-negative donors predominantly expressed c (71.8%), followed by e (65.38%), C (15.38%), and E (1.28%) (Table 5).

**Table 5:** Antigen frequency of Rh (C, c, E, e) in Rh (D)-positive and Rh (D)-negative donors (n = 1,000)

Rh(D) status		C	c	E	e	Total
Rh(D) positive	N	858	520	172	919	922
	%	93.06%	56.39%	18.66%	99.67%	100%
Rh(D) negative	N	12	56	1	51	78
	%	15.38%	71.80%	1.28%	65.38%	100%

The most common phenotype observed was Dce/Dce (R1R1) at 44.2%, followed by Dce/dce (R1r) at 32.1%. Rare phenotypes, such as DCE/DCE (RzRz) and others, were less frequently encountered (Table 6).

**Table 6:** Frequency of Rh phenotypes among donors (n = 1,000)

Rh phenotype	Number	Frequency (%)
Dce/Dce(R_1_R_1_)	442	44.2%
Dce/dce(R_1_r)	321	32.1%
DCe/DcE(R_1_R_2_)	103	10.35%
DCE/DcE(R_z_R_2_)	1	0.1%
DCE/DCE(R_z_R_z_)	0	0%
DcE/DcE(R_2_R_2_)	12	1.2%
dCe/dce(r^’^r)	9	0.9%
Dce/DCE(R_1_R_2_)	9	0.9%
DcE/dce(r’’r)	1	0.1%
dce/dce(rr)	48	4.8%
Dce/dce(R_2_r)	32	3.2%
Dce/dce(R_0_r)	22	2.2%
Total	1000	100%

The k antigen was detected in all donors (100%), while the K antigen was found in only 2.1%. The most common Kell phenotype was K-k+ (97.9%), followed by K+k+ (2.1%) (Table 7, Table 8).

**Table 7:** Frequency of Kell antigen (n = 1,000)

Kell antigen	Positive		Negative		Total
	Number	Frequency (%)	Number	Frequency (%)	
K	21	2.10%	979	97.9%	1,000
k	1000	100%	0	0%	1,000

**Table 8:** Frequency of Kell phenotypes (n = 1,000)

Kell phenotype	Number	Frequency (%)
K+k+	21	2.1%
K-k+	979	97.9%
K+k-	0	0%
Total	1,000	100%

Microscopic observations

**Figure 1:**
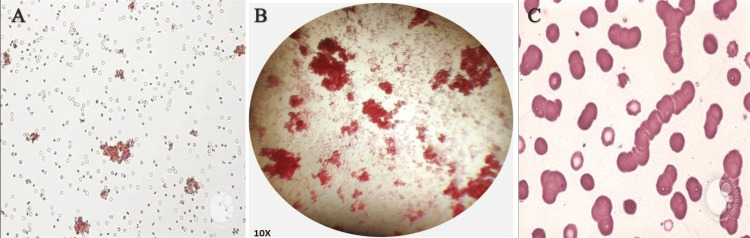
Microscopic images showing (A) weak agglutination, (B) mixed-field agglutination, and (C) rouleaux formation (A) Weak agglutination with dispersed small clusters of erythrocytes. (B) Mixed-field agglutination showing irregular clumping of red blood cells at 10× magnification. (C) Rouleaux formation, characterized by stacked erythrocytes resembling a coin-roll pattern, commonly observed in plasma protein abnormalities such as multiple myeloma or inflammatory conditions.

Microscopic examination revealed distinct patterns, including weak agglutination, mixed-field agglutination, and rouleaux formation, in specific donor samples (Figure 1).

## Discussion

Blood group polymorphisms show considerable variation across different populations due to genetic diversity and evolutionary selection pressures. These variations influence the phenotypic frequencies of blood groups across racial and ethnic groups, making the study of antigen distributions crucial for understanding transfusion compatibility and alloimmunization risks [22]. While Western nations benefit from advanced immunohematology technologies and comprehensive blood group databases, such resources remain limited in developing countries like India, highlighting the importance of studies like the present one [19].

In India, most blood banks rely on commercially available reagent red cell panels for antibody screening and identification. Since these panels are primarily derived from Western populations, they may not accurately reflect antigen frequencies in the Indian population, potentially leading to incompatible transfusions. Furthermore, routine cross-matching does not cover all clinically significant antigens, increasing the risk of alloimmunization. A practical solution to mitigate this issue would be to type voluntary blood donors for highly immunogenic antigens and establish a comprehensive donor registry. Such a database would facilitate the identification of antigen-negative blood units for alloimmunized patients, improving transfusion safety and efficiency.

In this study, the largest proportion of donors (342, 34.2%) belonged to blood group B, followed by group O (330, 33.0%), A (218, 21.8%), and AB (110, 11.0%). Among the donors, 922 (92.2%) were RhD positive, while only 78 (7.8%) were RhD negative.

These findings align with previous studies conducted in Ahmedabad and Mumbai, which reported similar ABO and Rh distributions [23,24]. In these studies, group O was consistently one of the most prevalent, while group AB was the least common. The RhD-positive frequency in our study (92.2%) closely matches the findings from Ahmedabad (95.05%) and Mumbai (94.61%) (Table 9).

**Table 9:** Distribution of ABO and Rh blood group phenotypes in different study populations

Author	Place of study	A (%)	B (%)	AB (%)	O (%)	Rh positive (%)	Rh negative (%)
Patel et al. (2012) [23]	Ahmedabad	21.95%	39.41%	7.85%	30.79%	95.05%	4.95%
Agrawal et al. (2014) [24]	Mumbai	22.88%	32.26%	7.74%	37.12%	94.61%	5.39%
Present study	Jamnagar	21.8%	34.2%	11.0%	33.0%	92.2%	7.8%

Among RhD-positive donors, the “e” antigen was the most prevalent (99.5%), followed by “C” (93.06%), “c” (56.39%), and “E” (18.66%). These findings are consistent with those reported by Kahar and Patel in South Gujarat [3] and Makroo et al. in New Delhi [19].

In the Kell blood group system, the “k” antigen was present in all donors (100%), while the “K” antigen was detected in 2.1% (Table 10). The most common Kell phenotype was K-k+ (97.9%), followed by K+k+ (2.1%), a distribution that aligns with previous Indian studies (Table 11).

**Table 10:** Distribution of Kell antigen variants in different study populations

Author	Place of study	C (%)	E (%)	e (%)	K (%)	k (%)
Makroo et al. (2013) [19]	New Delhi	87%	20.0%	98.0%	3.5%	99.9%
Kahar and Patel (2014) [3]	South Gujarat	81.74%	21.74%	100.0%	6.09%	100.0%
Present study	Jamnagar	93.06%	18.66%	99.67%	2.1%	100.0%

**Table 11:** Distribution of Kell phenotypes in different study populations

Author	Place of study	K+k+ (%)	K-k+ (%)	K+k- (%)
Makroo et al. (2013) [19]	New Delhi	3.50%	96.60%	0.00%
Kahar and Patel (2014) [3]	South Gujarat	6.09%	93.91%	0.00%
Present study	Jamnagar	2.1%	97.9%	0.00%

Our results align with previous studies conducted across various regions of India. Kahar and Patel [3] conducted one of the earliest studies on blood group phenotypic frequencies among donors in South Gujarat using the traditional tube method. Their extended Rh system antigen typing revealed that the “e” antigen was present in 100% of donors, followed by “C” (81.74%). Similar to our findings, the K-k+ phenotype in the Kell system was the most prevalent.

Thakral et al. [18] reported comparable Rh antigen frequencies, with the “e” antigen being the most common (98.3%) and the “E” antigen the least common (17.9%). Their findings on Kell antigen frequencies (K+k+ = 5.68%, K-k+ = 94.32%) were also consistent with the present study.

Makroo et al. [19] observed similar Rh phenotypic frequencies, with DCe/DCe (R1R1; 42.6%) being the most common and DCE/DCE (RzRz; 0.0%) the least common (Table 12).

**Table 12:** Distribution of Rh phenotypes in different study populations

Author	Place of study	R1R1 (%)	R1r (%)	R1R2 (%)	RzR2 (%)	RzRz (%)	R2R2 (%)	r’r (%)	R1R2 (%)	r’’r (%)	rr (%)	R2r (%)	R0r (%)
Makroo et al. (2013) [19]	New Delhi	42.6%	32.2%	14.5%	1.1%	0.0%	0.8%	0.3%	0.5%	0.2%	4.6%	0.1%	1.3%
Present study	Jamnagar	44.2%	32.1%	10.35%	0.1%	0.0%	1.2%	0.9%	0.9%	0.1%	4.8%	3.2%	2.2%

Our results are also consistent with those of Sarkar et al. [20], who reported the highest prevalence of “e” antigens (98.42%) and the lowest prevalence of “E” antigens (26.55%) in their study population.

Understanding the distribution of Rh and Kell antigens is essential for transfusion medicine, with significant implications for both clinical practice and research. Establishing a database of antigen-typed donors can enhance transfusion safety by ensuring compatibility for alloimmunized patients, particularly those in need of frequent transfusions. Moreover, studies on blood group antigen distribution offer valuable insights into population genetics, contributing to a deeper understanding of genetic diversity and inheritance patterns. These findings can also inform the development of region-specific serological panels, reducing reliance on commercially available Western-derived panels and improving transfusion compatibility within the Indian population. In addition to transfusion medicine, blood group polymorphisms have important medicolegal applications, including in forensic investigations and paternity testing, further emphasizing their relevance in both healthcare and legal contexts [25-27].

Limitations

This study has several limitations. First, it was conducted in a single geographic region, which may limit the generalizability of the findings to other regions of India. The extreme gender imbalance (99.7% male) could skew the results, potentially overlooking gender-specific patterns. Furthermore, the study relied on voluntary blood donors, which may not reflect the overall population distribution. The use of commercially available reagent red cell panels, primarily derived from Western populations, may lead to an underestimation of certain antigen frequencies that are unique to the Indian population. Lastly, the study did not include rare blood group phenotypes, which could be critical in specific clinical scenarios.

Recommendations

Future studies should aim to include a larger, more diverse sample from multiple geographic regions to enhance the generalizability of the findings. Developing region-specific red cell panels would better capture the antigenic diversity unique to the Indian population. Additionally, establishing a national database of blood group antigen frequencies and phenotypes would be invaluable in improving transfusion practices. Research focusing on rare blood group phenotypes and their clinical implications should also be prioritized. Collaborative efforts among blood banks and research institutions are encouraged to standardize methodologies and share data, ultimately benefiting patients across the country.

## Conclusions

This study underscores the importance of antigen-typing blood donors, particularly for Rh and Kell antigens, to enhance transfusion safety and reduce the risks of alloimmunization. The findings, consistent with other studies in India, highlight the need for comprehensive donor registries. Such registries would improve blood transfusion management, reduce alloimmunization, decrease the incidence of hemolytic disease in newborns, and prevent transfusion reactions. Routine extended Rh and Kell typing, combined with the development of robust antigen databases, is essential for optimizing patient care, ensuring compatibility, and efficiently managing blood resources in India.
